# Comparison of serum cytokines levels in normal-weight and overweight patients with first-episode drug-naïve major depressive disorder

**DOI:** 10.3389/fendo.2022.1048337

**Published:** 2022-10-27

**Authors:** Wenfan Gao, Yayun Xu, Jun Liang, Yanhong Sun, Yuanyuan Zhang, Feng Shan, Jinfang Ge, Qingrong Xia

**Affiliations:** ^1^ Affiliated Psychological Hospital of Anhui Medical University, Hefei, China; ^2^ Department of Pharmacy, Hefei Fourth People’s Hospital, Hefei, China; ^3^ Psychopharmacology Research Laboratory, Anhui Mental Health Center, Hefei, China; ^4^ Department of Science and Education, Anhui Clinical Research Center for Mental Disorders, Hefei, China; ^5^ Department of Epidemiology and Biostatistics, School of Public Health, Anhui Medical University, Hefei, China; ^6^ Inflammation and Immune Mediated Diseases Laboratory of Anhui Province, Anhui Institute of Innovative Drugs, Anhui Medical University, Hefei, China; ^7^ The Key Laboratory of Anti-inflammatory and Immune Medicines, Ministry of Education, Hefei, China; ^8^ School of Pharmacy, Anhui Medical University, Hefei, China

**Keywords:** cytokines, serum, overweight, major depressive disorder, body mass index

## Abstract

**Objective:**

Abnormal levels of blood cytokines have been demonstrated to be associated with both excess weight and major depressive disorder (MDD). However, few studies have addressed the direct effect of body mass index (BMI) on basal serum cytokines in individuals with first-episode drug-naïve MDD.

**Methods:**

A total of 49 patients with first-episode drug-naïve MDD were categorized into normal weight (18.5 ≤ BMI < 25 kg/m^2^) and overweight (25 ≤ BMI < 30 kg/m^2^) groups according to WHO-criteria. The severity of depressive symptoms was assessed using the 24-items Hamilton Depression Scale (HAMD-24). A total of 37 cytokines were measured using Multiplex Luminex Assays. The scores of HAMD-24 and the levels of serum cytokines between normal weight group and overweight group were compared. Multiple linear regression analysis was performed to evaluate the association between abnormal serum cytokines levels and group after adjusting for HAMD-24 scores. The correlation between BMI and the scores of HAMD-24 and the levels of serum cytokines was evaluated using Pearson correlation analysis.

**Results:**

The scores of HAMD-24 in overweight group were significantly higher than normal weight group (*t* = -2.930, *P* = 0.005). Moreover, the levels of IL-1α, IL-1RA, IL-3, CXCL10, TNF-α, and ICAM-1 in overweight patients with MDD were significantly higher than those in normal-weight patients with MDD (all *P* < 0.05). Furthermore, after adjustment for HAMD-24 scores, there was a significant correlation between abnormal serum cytokines levels (IL-1α, IL-1RA, IL-3, CXCL10, TNF-α, and ICAM-1) and group (all *P* < 0.05). Additionally, BMI was positively correlated to the serum levels of IL-1α (*r* = 0.428, *P* = 0.002), IL-3 (*r* = 0.529, *P* < 0.001), IL-6 (*r* = 0.285, *P* = 0.050), IL-10 (*r* = 0.423, *P* = 0.003), IL-12 (*r* = 0.367, *P* = 0.010), IL-15 (*r* = 0.300, *P* = 0.036), CXCL10 (*r* = 0.316, *P* = 0.030), TNF-α (*r* = 0.338, *P* = 0.021), and ICAM-1 (*r* = 0.440, *P* = 0.002) in MDD patients.

**Conclusions:**

These results provide direct evidence, probably for the first time, that overweight may be associated with several serum cytokines in patients with first-episode drug-naïve MDD. The underlying mechanisms are unclear and require further investigation.

## Introduction

Depression and obesity often occur comorbidly, and their association has been reported repeatedly (1, 2). Several lines of evidence indicate a bidirectional relationship between depression and obesity. It has been reported that individuals with obesity are 32% more likely to experience depression compared with adults with normal weight (3) and vice versa, individuals with depression are 58% more likely to be obese compared with individuals without depression (4). Although the exact pathophysiological mechanism that links depression and obesity is yet to be fully elucidated, aberrant expression of cytokines in periphery is thought to be one of the comorbid mechanisms.

Many evidences confirmed a close relationship between cytokines and MDD. Recently, a cumulative meta-analysis reported higher levels of interleukin-6 (IL-6) and C-reactive protein (CRP) in patients with MDD compared to non-depressive individuals (5). Moreover, depressed patients who are resistant to conventional antidepressants had higher concentrations of peripheral blood levels of pro-inflammatory cytokines (6, 7). Furthermore, alterations in peripheral cytokine levels were reported to be associated with antidepressant treatment outcomes in MDD (8). These findings suggest that peripheral cytokines are implicated in the pathophysiology of depression and may hold significant promise as potential treatment target for depressive symptoms.

Adipose tissue is now recognized as an active endocrine organ expressing and secreting a variety of cytokines that play parts in the pathogenesis of many obesity-related diseases (9, 10). Obesity can be regarded as a state of chronic subclinical inflammation characterized by increased expression of proinflammatory cytokines, adipokines, and chemokines (11, 12). It has been demonstrated that plasma levels of pro-inflammatory cytokines such as IL-6, tumor necrosis factor-α (TNF-α), and IL-1β were significantly higher in overweight individuals than normal-weight individuals (13, 14). Considering the dysfunctional balance of cytokines in overweight or obesity, taken together the fact that abnormal level of blood cytokines may be both a causal mechanism and potential treatment target for depressive symptoms, it is crucial to understand to what extent weight gain contribute to the aberrant cytokine expression observed in patients with MDD.

In the present study, we examined the serum levels of multiple cytokines in patients with first-episode drug-naïve MDD, with the aim to analyze the direct effect of BMI on basal serum cytokines in patients with MDD. A total of 49 patients with MDD were categorized into normal weight (18.5 ≤ BMI < 25 kg/m^2^) and overweight (25 ≤ BMI < 30 kg/m^2^) groups. A total of 37 cytokines in patients with MDD were measured and the 24-items Hamilton Depression Scale (HAMD-24) was used to estimate the severity of depressive symptoms. Subsequently, the levels of serum cytokines between normal-weight patients with MDD and overweight patients with MDD were compared. The correlation between BMI and the scores of HAMD-24 and the levels of serum cytokines was evaluated using Pearson correlation analysis.

## Materials and methods

### Study design and participants

This study was conducted at Anhui Mental Health Center between August 2020 and June 2022. Fifty-three patients with first-episode drug-naïve MDD were diagnosed by trained psychiatrists according to the Diagnostic and Statistical Manual for Psychiatric Disorders-Fifth Version (DSM-V). Common criteria for patient inclusion and exclusion were shown in **Table 1**. A total of 49 patients with first-episode drug-naïve MDD were enrolled and categorized into normal weight (18.5 ≤ BMI < 25 kg/m^2^) and overweight (25 ≤ BMI < 30 kg/m^2^) groups according to WHO-criteria. Consecutive sampling technique was used to select the participants. All subjects were ethnic Han Chinese living in Anhui province. In the present study, 34 women and 15 men were enrolled, which is consistent with the gender difference in MDD incidence (15, 16). The Chinese version of HAMD-24, which has good reliability with Cronbach’s alpha value of 0.714 (17), was used to evaluate the severity of depressive symptoms in all participants. This procedure was approved by the ethics committee of the Anhui Mental Health Center (registration number HFSY-IRB-PJ-XQR-2020001) and was conducted according to the principles of the Declaration of Helsinki. Informed consent was obtained from all the participants.

**Table 1:** Common criteria for patient inclusion and exclusion.

Inclusion criteria	Exclusion criteria
(1) being between the ages of 18-65	(1) current or lifetime history of major neurological disorders including Alzheimer’s disease, amyotrophic lateral sclerosis, ischemia, trauma, hepatic encephalopathy, Down’s syndrome, autism, multiple sclerosis, brain neoplasms, Parkinson’s disease and epilepsy
(2) meeting DSM-V criteria for depression	(2) current or lifetime history of other psychiatric disorders including anxiety, schizophrenia, bipolar disorder, obsessive-compulsive disorder, alcohol and substance abuse, and attention-deficit hyperactivity disorder
(3) Hamilton Depression Rating Scale-24 (HAMD-24) scores higher than 20	(3) current or lifetime history of chronic infections, inflammatory and immune disorders including rheumatoid arthritis, inflammatory bowel disease, and nephrotic syndrome, systemic lupus erythematosus, multiple sclerosis, autoimmune type I diabetes, asthma, sepsis, pulmonary fibrosis, primary biliary cirrhosis, autoimmune myasthenia gravis and stroke
(4) receiving no treatment with antidepressants, anti-inflammatory agents or other psychotropic drugs in the previous 3 months	(4) currently receiving anti-inflammatory treatment

### Blood sample collection and measurement of serum cytokines

The blood samples from the subjects was collected between 7:00 and 8:00 A.M., centrifuged at 1200 g for 10 min at 4°C. The supernatant was used as serum samples, which were maintained at -80°C until detection. The blood samples were collected at baseline before treatment. A total of 37 serum cytokines, including IL-1α (also called IL-1F1), IL-1β (also called IL-1F2), IL-1RA (also called IL-1F3), IL-2, IL-3, IL-4, IL-5, IL-6, IL-7, IL-8 (also called C-X-C motif chemokine ligand 8, CXCL8), IL-10, IL-12 (also called IL-23 p40), IL-12 p70, IL-13, IL-15, IL-16, IL-17C, IL-27, IL-31, C-C motif chemokine ligand 3 (CCL3; also called macrophage inflammatory protein 1α, MIP-1α), CCL4 (also called MIP-1β), CCL11 (also called eotaxin), CCL17 (also called thymus and activation regulated chemokine, TRAC), CCL26 (also called eotaxin-3), CXCL10 (also called interferon-inducible Protein 10, IP-10; cytokine responsive gene-2, CRG-2), vascular endothelial growth factor (VEGF), VEGF-C, VEGFR1 (also called Flt1), TNF-α, TNF-β (also called lymphotoxin), Tie-2, interferon-γ (IFN-γ), granulocyte-macrophage colony-stimulating factor (GM-CSF), fibroblast growth factor-basic (FGF basic, also called FGF2/bFGF), thymic stromal lymphopoietin (TSLP), intercellular cell adhesion molecule-1 (ICAM-1), and placenta growth factor (PIGF) were measured by the multiplex bead immunoassay (LXSAHM-10 and LXSAHM-27, R&D system for antibody detection, Shanghai Universal Biotech Co., Ltd) according to the manufacturer’s instructions.

### Statistical analysis

Statistical analysis was calculated using SPSS (version 17.0; IBM Corp., Armonk, NY, USA). The data are shown as mean ± standard error of the mean (SEM), and the statistical significance was set at *P* < 0.05. Student’s *t*-test for independent samples was used to compare the age, BMI, years of education, HAMD-24 scores, and serum cytokines between the two groups. Multiple linear regression analysis was performed to evaluate the association between abnormal serum cytokines levels and group after adjusting for HAMD-24 scores. Chi-squared test was used to determine the difference between the two groups with respect to sex and smoking status. The correlation between BMI and the scores of HAMD-24 and the levels of serum cytokines was evaluated using Pearson correlation analysis.

## Results

### Demographic and clinical characteristics of the participants

**Table 2** summarizes the demographic and clinical characteristics of normal-weight patients with MDD (n = 34) and overweight patients with MDD (n = 15). There were no significant differences in age, sex, years of education, or smoking status between the two groups (all *P* > 0.05; **Table 2**). The scores of HAMD-24 in overweight group were significantly higher than normal weight group (*t* = -2.930, *P* = 0.005).

**Table 2:** Demographic and clinical characteristics of normal-weight patients with MDD and overweight patients with MDD.

Variables	Normal weight	Overweight	*t*/*χ^2^*	*P*
Age	35.29 ± 2.51	42.13 ± 4.04	-1.474	0.147
Sex (F/M)	24/10	10/5	0.075	0.784
BMI (kg/m^2^)	21.40 ± 0.29	26.71 ± 0.29	-13.157	< 0.001
Education (years)	10.41 ± 0.93	9.27 ± 1.39	0.682	0.499
Smoking status (Y/N)	3/31	3/12	1.210	0.271
HAMD-24	32.35 ± 1.26	40.33 ± 2.97	-2.930	0.005

### Differences of cytokine levels in serum between normal-weight patients with MDD and overweight patients with MDD

As shown in **Table 3**, the levels of IL-1α, IL-1RA, IL-3, CXCL10, TNF-α, and ICAM-1 in overweight patients with MDD were significantly higher than those in normal-weight patients with MDD (all *P* < 0.05). There were no significant differences in other cytokines levels including IL-1β, IL-2, IL-4, IL-5, IL-6, IL-7, IL-8, IL-10, IL-12, IL-12 p70, IL-13, IL-15, IL-16, IL-17C, IL-27, IL-31, CCL3, CCL4, CCL11, CCL17, CCL26, VEGF, VEGF-C, VEGFR1, Tie-2, IFN-γ, GM-CSF, FGF basic, TSLP, and PIGF between the two groups (all *P* > 0.05; **Table 3**).

**Table 3:** Comparison of serum cytokines between MDD patients without SI and MDD patients with SI.

Variables (pg/ml)	MDD without SI	MDD with SI	*t*	*P*
IL-1α (IL-1F1)	3.29 ± 0.41	6.67 ± 1.38	-2.342	0.032
IL-1β (IL-1F2)	9.59 ± 1.45	10.08 ± 2.53	-0.176	0.861
IL-1RA (IL-1F3)	957.06 ± 129.08	1736.02 ± 453.57	-2.190	0.034
IL-2	32.14 ± 5.18	38.28 ± 11.11	-0.574	0.569
IL-3	25.97 ± 0.71	32.11 ± 1.93	-3.727	0.001
IL-4	30.74 ± 4.80	46.22 ± 9.66	-1.605	0.115
IL-5	2.05 ± 0.15	2.08 ± 0.17	-0.109	0.914
IL-6	9.77 ± 2.60	19.44 ± 6.41	-1.676	0.101
IL-7	10.63 ± 1.07	10.88 ± 1.48	-0.131	0.897
IL-8 (CXCL8)	531.98 ± 107.49	672.35 ± 265.39	-0.589	0.558
IL-10	2.76 ± 0.49	4.83 ± 1.06	-1.787	0.090
IL-12 (IL-23 p40)	216.43 ± 10.20	252.71 ± 27.34	-1.534	0.132
IL-12 p70	44.64 ± 10.35	27.46 ± 5.57	1.067	0.291
IL-13	232.07 ± 7.78	258.86 ± 14.09	-1.788	0.081
IL-15	3.68 ± 0.39	5.33 ± 0.96	-1.602	0.126
IL-16	170.54 ± 12.66	197.41 ± 17.74	-1.206	0.234
IL-17C	11.19 ± 0.75	11.94 ± 1.45	-0.503	0.617
IL-27	307.96 ± 21.95	369.68 ± 53.88	-1.061	0.302
IL-31	29.94 ± 1.46	28.67 ± 2.48	0.464	0.645
CCL3 (MIP-1α)	595.29 ± 112.33	732.10 ± 155.84	-0.678	0.501
CCL4 (MIP-1β)	473.28 ± 78.81	527.31 ± 89.24	-0.398	0.693
CCL11 (Eotaxin)	117.31 ± 9.25	120.52 ± 14.44	-0.189	0.851
CCL17 (TRAC)	306.02 ± 21.04	393.72 ± 58.28	-1.415	0.174
CCL26 (Eotaxin-3)	14.29 ± 1.06	15.06 ± 2.18	-0.356	0.723
CXCL10 (IP-10/CRG-2)	17.50 ± 0.91	21.54 ± 1.47	-2.387	0.021
VEGF	108.29 ± 10.95	144.82 ± 24.89	-1.343	0.195
VEGF-C	1722.34 ± 105.82	1898.45 ± 170.97	-0.901	0.372
VEGFR1 (Flt1)	150.48 ± 8.56	174.36 ± 22.01	-1.011	0.325
TNF-α	4.13 ± 0.47	6.34 ± 0.98	-2.308	0.026
TNF-β (Lymphotoxin)	2.57 ± 0.14	2.90 ± 0.27	-1.184	0.243
Tie-2	16009.29 ± 1189.25	18947.80 ± 3515.53	-1.005	0.320
IFN-γ	15.58 ± 0.98	18.19 ± 1.59	-1.418	0.163
GM-CSF	3.37 ± 0.31	3.89 ± 0.47	-0.927	0.359
FGF basic (FGF2/bFGF)	9.14 ± 1.03	12.22 ± 2.77	-1.043	0.311
TSLP	1.09 ± 0.05	1.14 ± 0.09	-0.489	0.627
ICAM-1 (CD54)	292954.01 ± 47435.41	674661.53 ± 116416.43	-3.036	0.007
PIGF	2.20 ± 0.12	2.46 ± 0.27	-1.006	0.319

Since the scores of HAMD-24 in overweight group were significantly higher than normal weight group, multiple linear regression analysis was performed to evaluate the association between abnormal serum cytokines levels and group after adjusting for HAMD-24 scores. As shown in **Table 4**, after adjustment for HAMD-24 scores, there was still a significant correlation between abnormal serum cytokines levels (IL-1α, IL-1RA, IL-3, CXCL10, TNF-α, and ICAM-1) and group (all *P* < 0.05).

**Table 4:** Multiple linear regression analysis to determine the independent predictors (HAMD-24 scores and group) of abnormal serum cytokines levels.

Dependent variables	Independent variables	*β*	*t*	*P*
IL-1α	Constant	2.254	1.094	0.280
	HAMD-24 scores	-0.097	-1.666	0.103
	Group	4.155	3.530	0.001
IL-1RA	Constant	252.434	0.369	0.714
	HAMD-24 scores	-3.050	-0.158	0.875
	Group	803.302	2.055	0.046
IL-3	Constant	24.761	8.706	< 0.001
	HAMD-24 scores	-0.204	-2.569	0.014
	Group	7.827	4.641	< 0.001
CXCL10	Constant	11.599	3.643	0.001
	HAMD-24 scores	0.050	0.544	0.589
	Group	4.305	2.293	0.027
TNF-α	Constant	1.082	0.602	0.550
	HAMD-24 scores	0.024	0.449	0.656
	Group	2.276	2.042	0.048
ICAM-1	Constant	20584.486	0.102	0.919
	HAMD-24 scores	-4427.182	-0.785	0.437
	Group	416320.036	3.645	0.001

### Correlation between BMI and HAMD-24 scores and the serum levels of cytokines

**Figure 1:**
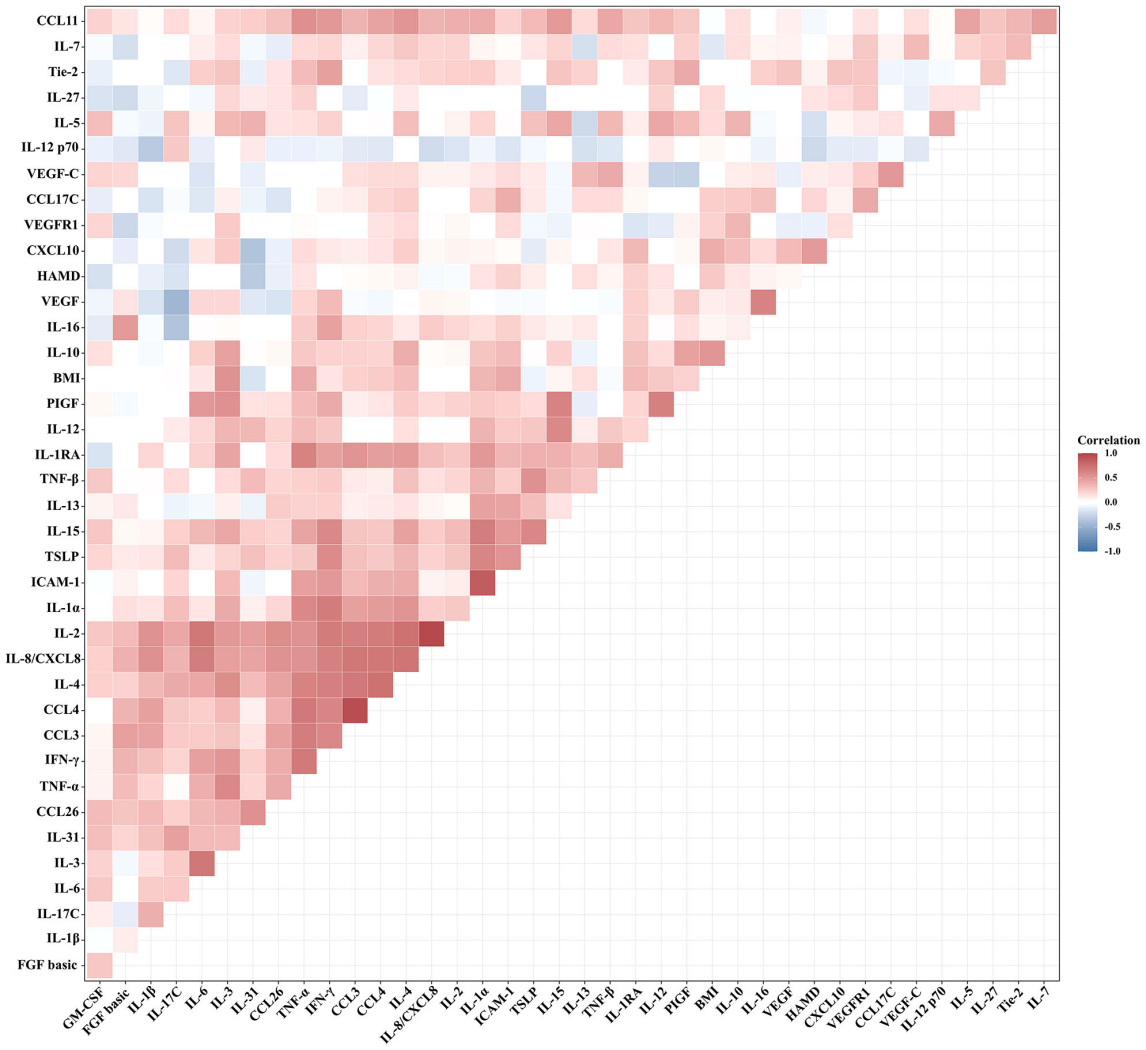
Correlation between BMI and HAMD-24 scores and the serum levels of cytokines in patients with MDD (Pearson correlation analysis).

The relationships between BMI and HAMD-24 scores and the serum levels of cytokines in patients with MDD were analyzed by Pearson correlation tests (**Figure 1**). There was no significant relationship between BMI and HAMD-24 scores (*r* = 0.236, *P* = 0.102). Among the serum cytokines, BMI was positively correlated to the serum levels of IL-1α (*r* = 0.428, *P* = 0.002), IL-3 (*r* = 0.529, *P* < 0.001), IL-6 (*r* = 0.285, *P* = 0.050), IL-10 (*r* = 0.423, *P* = 0.003), IL-12 (*r* = 0.367, *P* = 0.010), IL-15 (*r* = 0.300, *P* = 0.036), CXCL10 (*r* = 0.316, *P* = 0.030), TNF-α (*r* = 0.338, *P* = 0.021), and ICAM-1 (*r* = 0.440, *P* = 0.002).

## Discussion

To our knowledge, this is the first study to analyze the direct effect of BMI on basal serum cytokines in patients with first-episode drug-naïve MDD. Three main findings emerged from the present study. First, the scores of HAMD-24 in overweight group were significantly higher than normal weight group. Second, overweight patients with MDD showed higher serum levels of IL-1α, IL-1RA, IL-3, CXCL10, TNF-α, and ICAM-1. Third, BMI was positively correlated to the serum levels of IL-1α, IL-3, IL-6, IL-10, IL-12, IL-15, CXCL10, TNF-α, and ICAM-1 in MDD patients.

Several studies have evaluated the correlation between overweight or obesity and depression, and the results are conflicting. It has been reported that a positive relationship between overweight and depression scores was found in individuals from the United States and Europe (18–20). On the contrary, a negative correlation between BMI and depression scores were observed in individuals from Asia (21–23). In the present study, overweight patients with MDD showed higher scores of HAMD-24 compared to normal-weight patients with MDD, and no significant relationship between BMI and HAMD-24 scores in patients with first-episode drug-naïve MDD. The reasons for these conflicting relationships between BMI and depression scores remain unclear but might be due to the different races and variant designs (some for the general population and others for patients). Multicentric studies with larger sample sizes are required to validate these conflicting relationships.

IL-1α (a pro-inflammatory cytokine) and its inhibitor, IL-1RA (IL-1 receptor antagonist) belong to the IL-1 family. Previous studies have showed that increased IL-1α and IL-1RA concentrations in MDD patients compared to healthy controls (24, 25). Another study has indicated a positive correlation between increased BMI and elevated plasma level of IL-1α in obese women (26). IL-1RA can prevent the binding of IL-1α and IL1β to IL-1R1, and is reported to be upregulated mainly in adipose cells in obese individuals (27). Consistently, the results of the present study showed that the serum levels of IL-1α and IL-1RA were increased in overweight patients with MDD compared to normal-weight patients with MDD. Moreover, BMI was found to be positively correlated to the serum levels of IL-1α in patients with MDD.

IL-3 is a glycoprotein cytokine involved in the hematopoietic response to infection, immune response and inflammatory stimulation. Recently, a meta-analysis including 107 studies regrouping 5,166 patients with depression and 5,083 controls indicated that levels of IL-3 were significantly higher in patients with depression (28). Although IL-3 has been reported to be upregulated in obesity (29), few studies have reported differences in IL-3 levels in overweight and normal-weight patients with MDD. Our results firstly showed that the serum IL-3 levels were significantly elevated in overweight patients with MDD and were positively correlated with BMI.

CXCL10 is a member of the chemokine family secreted by various cell types, including monocytes, T cells, endothelial cells, and keratinocytes in response to secretion of IFN-γ and other proinflammatory cytokines (30). It has been reported that the serum levels of CXCL10 were elevated during depressive episodes, and this alteration correlated with increased depression severity (31, 32). More recent evidence indicated that peripheral blood levels of CXCL10 were significantly higher in obese subjects than in controls and significantly correlated with BMI (33). Combined with the results that the serum CXCL10 levels were significantly elevated in overweight patients with MDD and were positively correlated with BMI in patients with MDD in the present study, these findings provide more data linking peripheral CXCL10 to the obesity status in patients with MDD.

Elevated TNF-α levels are associated with a variety of conditions, including obesity and depression (34, 35). For example, recent meta-analyses have demonstrated very high concentrations of TNF-α in depressed patients as compared to healthy controls (36); elevated levels of TNF-α have been linked to poor antidepressant treatment response and (37, 38); and TNF-α inhibition can improve depressive-like behavior in clinical trials  (39). As a cytokine largely expressed in adipose tissue, TNF-α is elevated in obesity and may contribute to obesity-associated metabolic disease (40). Consistently, our results showed that the serum TNF-α levels were significantly elevated in overweight patients with MDD and were positively correlated with BMI.

ICAM-1, a member of the immunoglobulin protein superfamily, is a cell-surface glycoprotein that is overexpressed on the endothelial lumen in many pathological states (41). A recently published meta-analysis including 9,203 people with depression, found an association between higher ICAM-1 levels and depression (42). Another study has indicated that the ICAM-1 levels in MDD patients after a 3-day wash-out of antidepressants were significantly higher compared to healthy controls (37). Moreover, ICAM-1 level was found to be correlated with BMI and waist circumference in Mexican Americans (43). Similarly, the serum ICAM-1 levels were significantly elevated in overweight patients with MDD and were positively correlated with BMI in the present study.

It is noteworthy that IL-1β, IL-6, and IL-10 are the most frequently reported cytokines in depression and affective disorders. Several lines of evidence have demonstrated the involvement of IL-1β in MDD (44): (1) epidemiological data showed that levels of IL-1β in peripheral circulation and cerebrospinal fluid (CSF) of patients with MDD were increased; (2) antidepressants treatment could change the levels of IL-1β; (3) IL-1β administration could induce depression-like behaviors in rats. In the present study, there were no significant differences in serum IL-1β levels between normal-weight patients with MDD and overweight patients with MDD, suggesting that overweight may not be associated with serum IL-1β levels in patients with first-episode drug-naïve MDD. A updated meta-analysis based on 82 studies comprising 3212 participants with MDD and 2798 healthy controls has demonstrated that peripheral levels of IL-6 and IL-10 were elevated in patients with MDD compared to healthy controls while there was no significant change observed in the levels of IL-1β (25). In the present study, although there was no statistical difference, the serum IL-6 and IL-10 levels of overweight patients with MDD tended to be increased compared to normal-weight patients with MDD. Moreover, positive relationships were found between BMI and the serum levels of several interleukin family proteins including IL-6 and IL-10 in patients with first-episode drug-naïve MDD. Similarly, IL-6 levels were frequently elevated in obese subjects and positively correlated with obesity in human populations (45). Higher levels of IL-10 in overweight and obese subjects was correlated with BMI and the grade of abdominal obesity (13). Given that the sample size is relatively small, whether there are differences in the levels of IL-6 and IL-10 between normal-weight patients with MDD and overweight patients with MDD needs to be verified with large samples.

Additionally, positive relationships were also found between BMI and the serum levels of several interleukin family proteins including IL-12, and IL-15 in patients with first-episode drug-naïve MDD. Similarly, BMI was positively correlated to peripheral IL-12 and IL-15 in other studies. Specifically, overweight and obese subjects had higher levels of IL-12 than a normal-weight group, and this correlated with BMI and the grade of abdominal obesity (13). Another study revealed a positive relationship between circulating IL-15 concentration and fat mass in lean and obese participants (46). Taken together, these findings may link peripheral cytokines to the co-morbidity mechanism of depression and obesity. Given that this study is a cross-sectional study, the causal relationship between these cytokines and BMI in patients with MDD needs further studies to explore.

There are some limitations that should be considered. Firstly, the present study is a single-center study with a relatively small sample size, which might represent sampling bias. Secondly, in the absence of a control group (healthy subjects with normal weight), we had no baseline data for serum cytokines levels in the healthy population. Thirdly, we only used HAMD-24 to assess the severity of depressive symptom. In order to make it certain that the diagnosis based on HAMD-24 is properly validated, other scales should be used to assess the severity of depressive symptom. Fourthly, this study is a cross-sectional study, the causal relationship between these cytokines and BMI needs further studies to explore. Fifthly, CRP was not measured in the present study. However, numerous studies have reported that CRP was consistently associated with obesity and depression (47, 48).

In conclusion, the present study reveals that overweight patients with MDD showed higher levels of IL-1α, IL-1RA, IL-3, CXCL10, TNF-α, and ICAM-1 compared to normal-weight patients with MDD, and BMI was positively correlated to the serum levels of IL-1α, IL-3, IL-6, IL-10, IL-12, IL-15, CXCL10, TNF-α, and ICAM-1 in MDD patients. These findings provide evidence that overweight may be associated with several serum cytokines in patients with first-episode drug-naïve MDD. Since this study is a cross-sectional study and the causal relationship between these cytokines and BMI could not be determined, whether these cytokines are involved in the co-morbidity mechanism of depression and obesity, and whether they can be potential targets for treatment still need further studies to investigate.

## Data availability statement

The raw data supporting the conclusions of this article will be made available by the authors, without undue reservation.

## Ethics statement

The studies involving human participants were reviewed and approved by Anhui Mental Health Center. The patients/participants provided their written informed consent to participate in this study.

## Author contributions

WG, YX, JG, and QX conceived the study. YX, JG, and QX wrote the protocol. WG, JL, YS, YZ, and FS performed the analyses. WG and YX wrote the first draft. All authors read and commented the manuscript and agreed on the final version.

## Funding

This study was provided by the National Natural Science Foundation of China (81870403), Key Research and Development Program of Anhui Province (202004j07020001), Hefei Sixth cycle Key Medical Specialty, and Anhui Province Medical and Health Key Specialty Construction Project.

## Conflict of interest

The authors declare that the research was conducted in the absence of any commercial or financial relationships that could be construed as a potential conflict of interest.

## Publisher’s note

All claims expressed in this article are solely those of the authors and do not necessarily represent those of their affiliated organizations, or those of the publisher, the editors and the reviewers. Any product that may be evaluated in this article, or claim that may be made by its manufacturer, is not guaranteed or endorsed by the publisher.

## References

[1] Si T Yang K Lang X Dong X Wang N Zhang X. Prevalence and risk factors of overweight and obesity in Chinese patients with first-episode drug-naïve major depressive disorder. J Affect Disord (2021) 286:351–9. doi: 10.1016/j.jad.2021.01.037 33757648

[2] Chae W Nübel J Baumert J Gold S Otte C. Association of depression and obesity with c-reactive protein in Germany: A large nationally representative study. Brain Behav Immun (2022) 103:223–31. doi: 10.1016/j.bbi.2022.04.024 35491003

[3] Pereira-Miranda E Costa P Queiroz V Pereira-Santos M Santana M. Overweight and obesity associated with higher depression prevalence in adults: A systematic review and meta-analysis. J Am Coll Nutr (2017) 36:223–33. doi: 10.1080/07315724.2016.1261053 28394727

[4] Luppino FS de Wit LM Bouvy PF Stijnen T Cuijpers P Penninx BW. Overweight, obesity, and depression: A systematic review and meta-analysis of longitudinal studies. Arch Gen Psychiatry (2010) 67:220–9. doi: 10.1001/archgenpsychiatry.2010.2 20194822

[5] Haapakoski R Mathieu J Ebmeier K Alenius H Kivimäki M. Cumulative meta-analysis of interleukins 6 and 1β, tumour necrosis factor α and c-reactive protein in patients with major depressive disorder. Brain Behav Immun (2015) 49:206–15. doi: 10.1016/j.bbi.2015.06.001 PMC4566946 26065825

[6] Eller T Vasar V Shlik J Maron E. Pro-inflammatory cytokines and treatment response to escitalopram in major depressive disorder. Prog Neuropsychopharmacol Biol Psychiatry (2008) 32:445–50. doi: 10.1016/j.pnpbp.2007.09.015 17976882

[7] Amitai M Taler M Carmel M Michaelovsky E Eilat T Yablonski M. The relationship between plasma cytokine levels and response to selective serotonin reuptake inhibitor treatment in children and adolescents with depression and/or anxiety disorders. J Child Adolesc Psychopharmacol (2016) 26:727–32. doi: 10.1089/cap.2015.0147 26771135

[8] Liu JJ Wei YB Strawbridge R Bao Y Chang S Shi L. Peripheral cytokine levels and response to antidepressant treatment in depression: A systematic review and meta-analysis. Mol Psychiatry (2020) 25:339–50. doi: 10.1038/s41380-019-0474-5 31427752

[9] Galic S Oakhill J Steinberg G. Adipose tissue as an endocrine organ. Mol Cell Endocrinol (2010) 316:129–39. doi: 10.1016/j.mce.2009.08.018 19723556

[10] Ouchi N Parker J Lugus J Walsh K. Adipokines in inflammation and metabolic disease. Nat Rev Immunol (2011) 11:85–97. doi: 10.1038/nri2921 21252989 PMC3518031

[11] Dalamaga M Liu J. A chromatin remodeling checkpoint of diet-induced macrophage activation in adipose tissue. Metab Open (2022) 15:100204. doi: 10.1016/j.metop.2022.100204 PMC9386063 35990770

[12] Pischon N Heng N Bernimoulin J Kleber B Willich S Pischon T. Obesity, inflammation, and periodontal disease. J Dental Res (2007) 86:400–9. doi: 10.1177/154405910708600503 17452558

[13] Schmidt FM Weschenfelder J Sander C Minkwitz J Thormann J Chittka T. Inflammatory cytokines in general and central obesity and modulating effects of physical activity. PloS One (2015) 10:e0121971. doi: 10.1371/journal.pone.0121971 25781614 PMC4363366

[14] Medzhitov R. Origin and physiological roles of inflammation. Nature (2008) 454:428–35. doi: 10.1038/nature07201 18650913

[15] Kessler RC Berglund P Demler O Jin R Koretz D Merikangas KR. The epidemiology of major depressive disorder: results from the national comorbidity survey replication (NCS-r). JAMA (2003) 289:3095–105. doi: 10.1001/jama.289.23.3095 12813115

[16] Kessler R Berglund P Demler O Jin R Merikangas K Walters E. Lifetime prevalence and age-of-onset distributions of DSM-IV disorders in the national comorbidity survey replication. Arch Gen Psychiatry (2005) 62:593–602. doi: 10.1001/archpsyc.62.6.593 15939837

[17] Zheng YP Zhao JP Phillips M Liu JB Cai MF Sun SQ. Validity and reliability of the Chinese Hamilton depression rating scale. Br J Psychiatry J Ment Sci (1988) 152:660–4. doi: 10.1192/bjp.152.5.660 3167442

[18] Correia J Ravasco P. Weight changes in Portuguese patients with depression: Which factors are involved? Nutr J (2014) 13:117. doi: 10.1186/1475-2891-13-117 25516181 PMC4289568

[19] McIntyre R Konarski J Wilkins K Soczynska J Kennedy S. Obesity in bipolar disorder and major depressive disorder: Results from a national community health survey on mental health and well-being. Can J Psychiatry Rev Can Psychiatr (2006) 51:274–80. doi: 10.1177/070674370605100502 16986816

[20] Toups MS Myers AK Wisniewski SR Kurian B Morris DW Rush AJ. Relationship between obesity and depression: Characteristics and treatment outcomes with antidepressant medication. Psychosom Med (2013) 75:863–72. doi: 10.1097/PSY.0000000000000000 PMC3905462 24163386

[21] Kuriyama S Koizumi Y Matsuda-Ohmori K Seki T Shimazu T Hozawa A. Obesity and depressive symptoms in elderly Japanese: The tsurugaya project. J Psychosom Res (2006) 60:229–35. doi: 10.1016/j.jpsychores.2005.07.010 16516653

[22] Kim J Noh J Park J Kwon Y. Body mass index and depressive symptoms in older adults: a cross-lagged panel analysis. PloS One (2014) 9:e114891. doi: 10.1371/journal.pone.0114891 25501372 PMC4263712

[23] Luo H Li J Zhang Q Cao P Ren X Fang A. Obesity and the onset of depressive symptoms among middle-aged and older adults in China: evidence from the CHARLS. BMC Public Health (2018) 18:909. doi: 10.1186/s12889-018-5834-6 30041632 PMC6057008

[24] Cizza G Marques AH Eskandari F Christie IC Torvik S Silverman MN. Elevated neuroimmune biomarkers in sweat patches and plasma of premenopausal women with major depressive disorder in remission: the POWER study. Biol Psychiatry (2008) 64:907–11. doi: 10.1016/j.biopsych.2008.05.035 PMC2610843 18657799

[25] Köhler C Freitas TH Maes M de Andrade NQ Liu CS Fernandes BS. Peripheral cytokine and chemokine alterations in depression: A meta-analysis of 82 studies. Acta Psychiatr Scand (2017) 135:373–87. doi: 10.1111/acps.12698 28122130

[26] Ghanbari M Momen Maragheh S Aghazadeh A Mehrjuyan SR Hussen BM Abdoli Shadbad M. Interleukin-1 in obesity-related low-grade inflammation: From molecular mechanisms to therapeutic strategies. Int Immunopharmacol (2021) 96:107765. doi: 10.1016/j.intimp.2021.107765 34015596

[27] Juge-Aubry CE Somm E Giusti V Pernin A Chicheportiche R Verdumo C. Adipose tissue is a major source of interleukin-1 receptor antagonist: upregulation in obesity and inflammation. Diabetes (2003) 52:1104–10. doi: 10.2337/diabetes.52.5.1104 12716739

[28] Osimo E Pillinger T Rodriguez I Khandaker G Pariante C Howes O. Inflammatory markers in depression: A meta-analysis of mean differences and variability in 5,166 patients and 5,083 controls. Brain Behav Immun (2020) 87:901–9. doi: 10.1016/j.bbi.2020.02.010 PMC7327519 32113908

[29] Dushnicky M Nazarali S Mir A Portwine C Samaan M. Is there a causal relationship between childhood obesity and acute lymphoblastic leukemia? A Rev Cancers (2020) 12:3082. doi: 10.3390/cancers12113082 PMC7690432 33105727

[30] Metzemaekers M Vanheule V Janssens R Struyf S Proost P. Overview of the mechanisms that may contribute to the non-redundant activities of interferon-inducible CXC chemokine receptor 3 ligands. Front Immunol (2017) 8:1970. doi: 10.3389/fimmu.2017.01970 29379506 PMC5775283

[31] Siwek M Sowa-Kućma M Styczeń K Misztak P Nowak RJ Szewczyk B. Associations of serum cytokine receptor levels with melancholia, staging of illness, depressive and manic phases, and severity of depression in bipolar disorder. Mol Neurobiol (2017) 54:5883–93. doi: 10.1007/s12035-016-0124-8 27660275

[32] Rosenblat J McIntyre R. Bipolar disorder and immune dysfunction: Epidemiological findings, proposed pathophysiology and clinical implications. Brain Sci (2017) 7:144. doi: 10.3390/brainsci7110144 PMC5704151 29084144

[33] Moreno B Hueso L Ortega R Benito E Martínez-Hervas S Peiro M. Association of chemokines IP-10/CXCL10 and I-TAC/CXCL11 with insulin resistance and enhance leukocyte endothelial arrest in obesity. Microvasc Res (2022) 139:104254. doi: 10.1016/j.mvr.2021.104254 34534571

[34] Chung H Choi K. Adipokines and myokines: A pivotal role in metabolic and cardiovascular disorders. Curr Med Chem (2018) 25:2401–15. doi: 10.2174/0929867325666171205144627 29210643

[35] Pegoretti V Baron W Laman J Eisel U. Selective modulation of TNF-TNFRs signaling: Insights for multiple sclerosis treatment. Front Immunol (2018) 9:925. doi: 10.3389/fimmu.2018.00925 29760711 PMC5936749

[36] Ma K Zhang H Baloch Z. Pathogenetic and therapeutic applications of tumor necrosis factor-α (TNF-α) in major depressive disorder: A systematic review. Int J Mol Sci (2016) 17:733. doi: 10.3390/ijms17050733 PMC4881555 27187381

[37] Haapakoski R Ebmeier K Alenius H Kivimäki M. Innate and adaptive immunity in the development of depression: An update on current knowledge and technological advances. Prog Neuropsychopharmacol Biol Psychiatry (2016) 66:63–72. doi: 10.1016/j.pnpbp.2015.11.012 26631274 PMC4736094

[38] Yao L Pan L Qian M Sun W Gu C Chen L. Tumor necrosis factor-α variations in patients with major depressive disorder before and after antidepressant treatment. Front Psychiatry (2020) 11:518837. doi: 10.3389/fpsyt.2020.518837 33364982 PMC7750423

[39] Raison C Rutherford R Woolwine BJ Shuo C Schettler P Drake DF. A randomized controlled trial of the tumor necrosis factor antagonist infliximab for treatment-resistant depression: the role of baseline inflammatory biomarkers. JAMA Psychiatry (2013) 70:31–41. doi: 10.1001/2013.jamapsychiatry.4 22945416 PMC4015348

[40] Rydén M Arner P. Tumour necrosis factor-alpha in human adipose tissue – from signalling mechanisms to clinical implications. J Internal Med (2007) 262:431–8. doi: 10.1111/j.1365-2796.2007.01854.x 17875179

[41] Müller N. The role of intercellular adhesion molecule-1 in the pathogenesis of psychiatric disorders. Front Pharmacol (2019) 10:1251. doi: 10.3389/fphar.2019.01251 31824303 PMC6883971

[42] van Agtmaal M Houben A Pouwer F Stehouwer C Schram M. Association of microvascular dysfunction with late-life depression: A systematic review and meta-analysis. JAMA Psychiatry (2017) 74:729–39. doi: 10.1001/jamapsychiatry.2017.0984 PMC5710252 28564681

[43] Kent JW Comuzzie AG Mahaney MC Almasy L Rainwater DL VandeBerg JL. Intercellular adhesion molecule-1 concentration is genetically correlated with insulin resistance, obesity, and HDL concentration in Mexican americans. Diabetes (2004) 53:2691–5. doi: 10.2337/diabetes.53.10.2691 15448102

[44] Mota R Gazal M Acosta BA de Leon PB Jansen K Pinheiro RT. Interleukin-1β is associated with depressive episode in major depression but not in bipolar disorder. J Psychiatr Res (2013) 47:2011–4. doi: 10.1016/j.jpsychires.2013.08.020 24074516

[45] Ambrósio G Kaufmann FN Manosso L Platt N Ghisleni G Rodrigues ALS. Depression and peripheral inflammatory profile of patients with obesity. Psychoneuroendocrinology (2018) 91:132–41. doi: 10.1016/j.psyneuen.2018.03.005 29550676

[46] Pérez-López A Valadés D Vázquez Martínez C de Cos Blanco A Bujan J García-Honduvilla N. Serum IL-15 and IL-15Rα levels are decreased in lean and obese physically active humans. Scand J Med Sci Sports (2018) 28:1113–20. doi: 10.1111/sms.12983 28940555

[47] Chen MH Hsu JW Huang KL Tsai SJ Su TP Li CT. Role of obesity in systemic low-grade inflammation and cognitive function in patients with bipolar I disorder or major depressive disorder. CNS Spectr (2021) 26:521–7. doi: 10.1017/S1092852920001534 32594934

[48] Frank P Jokela M Batty G Cadar D Steptoe A Kivimäki M. Association between systemic inflammation and individual symptoms of depression: A pooled analysis of 15 population-based cohort studies. Am J Psychiatry (2021) 178:1107–18. doi: 10.1176/appi.ajp.2021.20121776 34645276

